# Follow-up effects of a brief Good Psychiatric Management treatment for borderline personality disorder

**DOI:** 10.1186/s40479-026-00357-z

**Published:** 2026-07-15

**Authors:** José Blanco Machinea, Loris Grandjean, Hélène Beuchat, Stéphane Kolly, Philippe Conus, Yves de Roten, Jérémie André, Hervé Tissot, Ueli Kramer

**Affiliations:** 1 Department of Psychiatry, University Institute of Psychotherapy, University of Lausanne and Lausanne University Hospital, Route de Cery 1, Prilly-Lausanne, CH-1008 Switzerland; 2 General Psychiatry Service, Department of Psychiatry, Lausanne University Clinic and University of Lausanne, Lausanne, Switzerland; 3 Department of Psychology, University of Windsor, Windsor, Canada

**Keywords:** Borderline personality disorder, Follow-up, Outcome research, Good psychiatric management

## Abstract

**Background:**

Borderline personality disorder (BPD) is among the most frequent, debilitating, and lethal mental conditions. Treatment for patients with BPD involves structured psychotherapy, but such treatment is often not readily available due to imbalances in supply and demand for care. Brief treatments that are available as first-line interventions may address this delay in care. No controlled study has assessed the effectiveness of brief psychiatric interventions, such as 10 sessions of Good Psychiatric Management (GPM) over 12 months follow-up, in comparison with treatment as usual. Whereas most psychotherapy studies in patients with BPD focus on symptom reduction at the end of treatment, more research is needed on the patients’ symptom levels after one year.

**Methods:**

The present study compares brief psychiatric treatment (4 months of GPM) with treatment as usual (4 months of TAU). Participants (*N* = 76 patients with BPD) undergo assessments at intake, after two months, at discharge (4 months) and 1 year follow-up (16 months after intake). At these four time-points, each patient was assessed using the clinician rated Zanarini BPD (ZAN-BPD) scale.

**Results:**

The study found brief GPM to show significantly greater symptom reduction in ZAN-BPD scores between intake of 4-month treatment and 12-month follow-up (16 months later), compared to treatment as usual.

**Conclusion:**

This study shows that after one year, the effects of a brief psychiatric treatment are strengthened in both groups. Additionally, brief GPM presents an advantage in helping BPD patients make further progress after the intervention has ended, which potentially helps diminish the societal burden of disease related to BPD symptomatology.

## Background

Borderline personality disorder (BPD) is a debilitating and sometimes lethal mental disorder. Patients who suffer from BPD often generate a heavy burden on health costs due to their need for emergency services, inpatient and outpatient treatments [1]. A recent meta-analysis shows that psychological treatments are the first-line treatments for symptoms associated with BPD [2]. Out of the 75 studies included in this metanalysis [2], 36 focused on treatments shorter or equal to 6 months and 32 with treatment duration ranging between 6 and 12 months, with unclear maintenance of their effects over longer periods of time. Traditionally, long-term treatment is proposed to patients with BPD (longer than a year), for good reasons, but changes are already visible after shorter timeframes. Additionally, even supply for first-step care for BPD is drastically inferior to the demand [3, 4]. This makes it a priority to verify the efficacy of easily trainable interventions which inevitably poses the question of the long-term impact of short-term treatments.

For symptom change to truly matter, we must verify if what was gained during the treatment is maintained or even strengthened after a significant amount of time. Some studies that incorporate a long-term follow-up assessment include Bateman and Fonaguy [5], which had a follow-up at 18 months, which compared Mentalization-Based Treatment with Structured Clinical Management regarding outcomes and functioning for BPD. Another study by McMain and colleagues [6], with follow-up at 2 years after intake, compared clinical outcomes and functioning after one year treatment of either Dialectical Behavioral therapy or Good Psychiatric Management (GPM), for BPD patients. It showed long-lasting positive effects of both treatments in long-term variants of DBT and GPM.

GPM is designed to address the gap of access to treatment for patients who suffer from BPD [7]. It is a clinically derived treatment that blends cognitive-behavioral and psychodynamic strategies. It emphasizes awareness of BPD functioning and interpersonal hypersensitivity and has shown encouraging results [7]. Recently, Kramer et al. [8] compared the effects of brief GPM (10 sessions) to TAU. The brief GPM intervention focused on BPD specific problems and understanding whereas the TAU intervention had to avoid discussing BPD specifics such as diagnostic, emotional or interpersonal factors. The main report of outcomes for this study described significant pre-post differences on average, which did not differ between the two conditions. However, a specific positive effect favoring GPM was found for the reduction in interpersonal problems [9].

The present study aims at determining the evolution of patients with BPD’s symptoms over the period ranging between intake and 16 months after intake, for patients having received brief GPM compared to TAU. We predict that both groups will remain stable between 4 months and 16 months after intake and that both groups will display differences in the symptoms subscales as their methods differ. Demonstrating effects over time, one year after treatment can potentially reveal some of the true impact the treatment has had on their psychosocial functioning.

## Methods

The present study was conducted between January 2019 and October 2023. It took place in a university psychiatry department outpatient, European French-speaking clinic. The study compared brief Good Psychiatric Management (GPM) specifically developed for patients with BPD [10] to treatment as usual (TAU), in a randomized control trial. With respect to the ethical guidelines, the competent ethics committee approved the study (NCT03717818, date of registration and project number: 2017–02167). The main results have been published [8] and the present study will look at change in scores between intake and the 1 year follow-up data.

### Participants, therapists and assessors

At intake, *N* = 76 patients were included into the study, (50 (66%) reported women), aged 18 to 60 (M = 33, SD = 8.26), randomized into two conditions: GPM = 35 (46%) and TAU = 41. Patients were made aware of which group they were assigned to.

There were *N* = 28 therapists, 23 psychiatrists and 5 psychologists with three years of clinical experience in psychiatry, on average. They had between 1 and 20 years of experience, averaging 4 years, and they all received at least one day of intensive workshop in GPM.

There were *N* = 4 psychologist researchers-assessors They were at the MPs, PhD or post-doc levels. All of them received comprehensive training in psychotherapy research methodology and the assessment procedures.

### Treatment

In the GPM condition, Six principles were implemented at every session: (a) therapist activity and structuring, (b) therapist support, (c) focus on life situations, work and relationship outside of the session, (d) therapist relationship interventions, (e) explicit expectation of change, and (f) therapist fostering patient’s accountability. The treatment included communication about the BPD diagnosis and co-morbidities, elaboration of links between problem areas (e.g., dependency, self-harming behavior and anger), interpersonal coherence model and emotion focus, the work on treatment goals and change, and a discussion of treatment-interfering behaviors [8]. The last session (4 months) involved the synthesis of the main achievements and a decision for next steps in the treatment program.

In the TAU condition, non-specific crisis management intervention, following minimal ethical standards and guidelines of good medical practice, was used. This was approved by the ethics committee as a non-specific treatment relying on common factors. Psychotropic medication was allowed. Discussion about the BPD diagnosis specifics were prohibited.

The therapists treated participants in both arms of the study. To ensure no overlap in the interventions, therapist adherence to GPM principles was assessed using Gunderson’s (2016; personal communication) questionnaire for adherence to GPM.

### Instrumenst

The Zanarini Rating Scale for Borderline Personality Disorder (ZAN-BPD [9]). It assesses 9 DSM-5 criteria from 0 to 4 for a total potential score of 36 (Cronbach’s alpha = 0.85). These nine items are also grouped into four sub-scales (affective, cognitive, impulsive and relationship symptoms). It is a continuous and clinician-administered scale. The mean score for a population suffering from BPD is 14.3 with a standard deviation of 6.8 [9]. The mean score for a non-BPD population is 5.2 with a standard deviation of 3.5 [9].

### Procedure

Participants were recruited at their intake (T1), where the therapist suggested the study. Soon after, the research team would contact them for a first interview where they would be randomized into either the GPM or TAU group, after which, the first assessment would properly start. The second interview happened after 2 months of treatment and the third interview happened after discharge (T3: 4 months). We then assessed all participants again after one year for the follow up (T4). The patients answered the questions asked by the psychologist-researcher with the following the ZAN-BPD was administered and scored according to Zanarini (2003). The average inter-rater reliability at measured at T1 was ICC = 0.857. Additionally, as a secondary outcome, we looked at the patients’ medical files for the year following T3 to find any ongoing therapy or hospitalization within the university clinic, after discharge to gage post-treatment health services use. This was operationalized into two dichotomic variables representing any consultation or hospitalization for the patient, in the time between discharge and 16-month follow-up (Consult: Yes – No / Hosp. Yes - No).

### Statistical analyses

Outcome was defined as symptom levels at 12-month follow-up. Analyses on primary outcome were carried out on the full sample (including all randomized individuals).

The main hypotheses were tested using structural equation models estimated in Mplus [11]. A Latent Growth Curve Model [12] was separately estimated to for the for ZAN-BPD dimensional scores, as well as for the total score, to model the evolution of the variables between T1 and T4. In order to limit the complexity of the models, we chose to model a linear evolution. The models thus included two latent variables, representing the baseline score (the Intercept variable) and the change in scores across time points (the Slope variable). For each model, we used multigroup analyses to compare the evolution of ZAN-BPD scores of patients randomized to GPM or TAU. The models were estimated using robust maximum likelihood estimation (MLR) in combination with multiple imputation (*n* = 50 imputed datasets) to handle missing data. Multiple imputation was required to offset the relatively high amount of dropouts. Parameter estimates and standard errors were pooled across imputations using Rubin’s rules. Model fit was evaluated using multiple indices, including the chi-square statistic (χ²), the root mean square error of approximation (RMSEA), the comparative fit index (CFI), the Tucker–Lewis index (TLI), and the standardized root mean square residual (SRMR). Because the MLR estimator applies a scaling correction to the chi-square statistic that varies across imputed datasets, a single pooled chi-square value is not available. Accordingly, model fit was assessed based on the empirical distribution of fit indices across imputations, including their mean values and percentile information, rather than on a single inferential chi-square test. The chi-square statistic was therefore interpreted diagnostically, by comparing the empirical distribution across imputations to the theoretical chi-square distribution with the corresponding degrees of freedom. In this context, chi-square values close to their corresponding degrees of freedom were taken as indicative of acceptable model fit. However, greater emphasis was placed on approximate fit indices when evaluating overall model fit. Criteria used to diagnose model fit on approximate fit indices were CFI > 0.95 (0.90 for acceptable fit), TLI > 0.95 (0.90 for acceptable fit), RMSEA < 0.06 (0.08 for acceptable fit), and SRMR < 0.08 [13].

For our secondary outcome we calculated the chi-squared of both conditions’ uses of services with the “consult” and “hosp” dichotomized variables. SPSS29 [14] was used for the latter analyses.

## Results

### Multigroup comparison of the evolution of ZAN-BPD total scores

The LGCM comparing the evolution ZAN-BPD total scores in patients randomized to GPM or TAU demonstrated good fit across multiple imputation–adjusted fit indices (see Table 1). The mean chi-square value closely aligned with the expected distribution, with no imputed datasets exceeding the 95th percentile. Approximate fit indices indicated good fit (RMSEA = 0.046; CFI = 0.97; TLI = 0.99), although the SRMR was somewhat elevated (SRMR = 0.096). Overall, the results suggest that the model provides an adequate to good representation of the data.

**Table 1:** Model fits indices for growth curve models comparing the evolution of ZAN-BPD scores (total and subscales scores) between the GPM and the TAU groups

Model	χ²	df	χ²/df	CFI	TLI	RMSEA	SRMR
Total score	10.599	10	1.059	0.970	0.986	0.046	0.096
Cognitive problems score	11.325	10	1.133	0.960	0.975	0.058	0.097
Affective problems score	13.074	10	1.307	0.928	0.940	0.076	0.103
Impulsivity score	16.012	10	1.601	0.851	0.821	0.116	0.118
Relationship score	19.437	10	1.944	0.827	0.792	0.149	0.123

Note. All indices were averaged from the results of the model estimation in 50 datasets (missing data imputation procedure). CFI = comparative fit index; TLI = Tucker-Lewis index; RMSEA = root mean square error of approximation; SRMR = standardized root mean square residual

The parameter estimates in the GPM group showed that the means for the Intercept and Slope variables were significant (respectively *M* = 15.850, *p* < 0.001 and *M* = -2.278, *p* < 0.001), which indicated that baseline and change scores were significantly different from zero. The reduction in ZAN-BPD total score for GPM patients was of more than two points on average between each time point. The variance of the Intercept variable was also significant (*s*^*2*^ = 32.586, *p* = 0.007), which suggested that GPM patients’ scores at baseline were significantly heterogeneous. The variance of the Slope variable, on the contrary, was not significant (*s*^*2*^ = 1.243, *p* = 0.541), suggesting that ZAN-BPD scores decreased in the same way for most patients. Concerning the TAU group, the parameter estimates showed that the means for the Intercept and Slope variables were both significant (respectively *M* = 13.271, *p* < 0.001 and *M* = -1.112, *p* = 0.005), which showed that baseline and change scores were significantly different from zero. The decrease in ZAN-BPD for TAU patients was of more than one point on average between each time point. The variance of the Intercept factor was also significant (*s*^*2*^ = 23.845, *p* = 0.033), which suggested that TAU patients’ scores at baseline were significantly heterogeneous. The variance of the Slope variable, on the contrary, was not significant (*s*^*2*^ = 2.395, *p* = 0.541), suggesting that ZAN-BPD scores decreased in the same way for most TAU patients. Comparing the evolution of the two groups, the results showed that, although ZAN-BPD scores decreased significantly in both groups, the decrease was significantly greater in the GPM group (*ΔM =* -1.166, *p* = 0.028).

### Multigroup comparison of the evolution of cognitive problems related to BPD

The LGCM comparing the evolution of ZAN-BPD scores on the “cognitive problems” dimension in patients randomized to GPM or TAU showed good overall fit under multiple imputation. The mean chi-square value, χ² (10) = 11.33, was close to the expected value, with observed proportions across the chi-square distribution largely consistent with theoretical expectations and only minimal tail deviations. Approximate fit indices supported this conclusion, with RMSEA indicating acceptable to close fit (RMSEA = 0.058) and high incremental fit indices (CFI = 0.960; TLI = 0.975). The SRMR was slightly elevated (SRMR = 0.097), suggesting modest residual misfit. Overall, the results indicate that the model provides an adequate to good representation of the data.

The estimation of the parameters in the GPM group showed that the means for the Intercept and Slope variables were significant (respectively *M* = 3.458, *p* < 0.001 and *M* = -0.482, *p* < 0.001 (see Table 2)), which indicated that baseline and change scores were significantly different from zero. The reduction in cognitive problems score for GPM patients was almost of half a point on average between each time point. The variance of the Intercept factor was also significant (*s*^*2*^ = 3.395, *p* = 0.007), which suggested that GPM patients’cognitive problems scores at baseline were significantly heterogeneous. The variance of the Slope variable, on the contrary, was not significant (*s*^*2*^ = 0.077, *p* = 0.781), suggesting that cognitive problems decreased in the same way for most patients. Concerning the TAU group, the parameter estimates showed that the means for the Intercept variable was significant (*M* = 2.663, *p* < 0.001), indicating that baseline scores were significantly different from zero. On the contrary, the mean of the Slope factor was not significant, suggesting no significant change for the TAU patients on cognitive problems. The variance of the Intercept and Slope variables were both significant (respectively *s*^*2*^ = 3.290, *p* = 0.005 and *s*^*2*^ = 0.375, *p* = 0.043), which suggested that TAU patients’ cognitive problems at baseline, as well as their rate of change between T1 and T4, were significantly heterogeneous. Comparing the evolution of the two groups, it should be underlined that, although cognitive problems decreased significantly only in the GPM group, the difference between the two groups was only close to the threshold of significance (*ΔM =* -0.298, *p* = 0.096).

**Table 2:** Results of parameter estimation of growth factors in growth curve models of ZAN-BPD total score, cognitive problems score, and affective problems score

Parameter	ZAN-BPD total score		ZAN-BPD cognitive problems		ZAN-BPD affective problems	
	TAU	GPM	TAU	GPM	TAU	GPM
Means						
Intercept	13.271 (0.967)***	15.850 (1.159)***	2.663 (0.317)***	3.458 (0.385)***	6.046 (0.412)***	7.406 (0.480)***
Slope	-1.112 (0.393)**	-2.278 (0.383)***	-0.184 (0.127)	-0.482 (0.132)***	-0.643 (0.194)**	-1.004 (0.183)***
Variances						
Intercept	23.845 (11.171)*	32.586 (12.027)**	3.290 (1.164)**	3.395 (1.250)**	2.926 (2.146)	5.331 (1.984)**
Slope	2.395 (1.804)	1.243 (2.033)	0.375 (0.185)*	0.077 (0.275)	0.675 (0.463)	0.521 (0.447)
Covariance						
Intercept–Slope	-5.534 (3.880)	-4.156 (3.344)	-0.943 (0.414)*	-0.485 (0.490)	-0.759 (0.760)	-0.669 (0.720)

Note. Displayed parameters are unstandardized means and standard errors (in parentheses). ZAN-BPD = Zannarini rating scale for borderline personality disorder; TAU = treatment as usual; GPM = general psychiatric management

* *p* < 0.05. ** *p* < 0.01. *** *p* < 0.001

### Multigroup comparison of the evolution of affective problems related to BPD

The LGCM comparing the evolution of ZAN-BPD scores on the “affective problems” dimension in patients randomized to GPM or TAU demonstrated adequate but not optimal fit under multiple imputation. The mean chi-square value, χ²(10) = 13.07, was moderately higher than expected, with a nontrivial proportion of imputed datasets exceeding the upper tail of the theoretical chi-square distribution, suggesting some degree of model misfit. Approximate fit indices were mixed, with RMSEA indicating acceptable fit (RMSEA = 0.076), whereas incremental fit indices were slightly below conventional cutoffs (CFI = 0.93; TLI = 0.94). The SRMR was elevated (SRMR = 0.103), pointing to residual misfit. Overall, the results only suggested a moderate fit, with evidence of some misspecification.

The estimation of the parameters in the GPM group showed that the means for the Intercept and Slope variables were significant (respectively *M* = 7.406, *p* < 0.001 and *M* = -1.004, *p* < 0.001 (see Table 2)), which indicated that baseline and change scores were significantly different from zero. The reduction in affective problems score for GPM patients was of one point on average between each time point. The variance of the Intercept factor was also significant (*s*^*2*^ = 5.331, *p* = 0.007), which suggested that GPM patients’ affective problems scores at baseline were significantly heterogeneous. The variance of the Slope variable, on the contrary, was not significant (*s*^*2*^ = 0.521, *p* = 0.244), suggesting that affective problems decreased in the same way for most patients. Concerning the TAU group, the estimation of the parameters showed that the means for the Intercept and Slope variables were significant (respectively *M* = 6.046, *p* < 0.001 and *M* = -0.643, *p* = 0.001), which indicated that baseline and change scores were significantly different from zero. The reduction in affective problems score for GPM patients was of more than half a point on average between each time point. The variance of the Intercept and Slope variables were both not significant (respectively *s*^*2*^ = 2.926, *p* = 0.173 and *s*^*2*^ = 0.675, *p* = 0.145), which suggested that TAU patients’ affective problems at baseline, as well as their rate of change between T1 and T4, were significantly similar for most patients. Comparing the evolution of the two groups, it should be underlined that, although affective problems decreased more in the GPM group as compared to the TAU group, this difference was not significant (*ΔM =* -0.361, *p* = 0.165). However, results showed a difference in baseline scores, with GPM patients presenting significantly more affective problems than patients in the TAU condition (*ΔM =* 1.360, *p* = 0.032).

### Multigroup comparison of the evolution of impulsivity related to BPD

The model exhibited poor overall fit under multiple imputation. The mean chi-square value, χ²(10) = 16.01, was substantially higher than expected, with a large proportion of imputed datasets falling in the upper tail of the theoretical chi-square distribution, indicating pronounced global misfit. Approximate fit indices consistently supported this conclusion, with RMSEA indicating poor fit (RMSEA = 0.116) and incremental fit indices well below conventional thresholds (CFI = 0.85; TLI = 0.82). The SRMR was also elevated (SRMR = 0.118), reflecting substantial residual misfit. Overall, these results suggest that the model does not adequately represent the data. Consequently, the parameter estimation of this model was untrustworthy and will not be presented here.

### Multigroup comparison of the evolution of relationship related to BPD

The LGCM comparing the evolution of ZAN-BPD scores on the “relationship” dimension in patients randomized to GPM or TAU demonstrated very poor overall fit under multiple imputation. The mean chi-square value, χ² (10) = 19.44, was markedly higher than expected, with a substantial proportion of imputed datasets falling in the upper tail of the theoretical chi-square distribution, indicating pronounced global misfit. Approximate fit indices consistently reflected poor fit, with a high (RMSEA = 0.149), low incremental fit indices (CFI = 0.83; TLI = 0.79), and an elevated (SRMR = 0.123). Collectively, these results indicate that the model fails to adequately represent the data and should be rejected. Consequently, the parameter estimation of this model was untrustworthy and will not be presented here.

### Multigroup comparison in post treatment services used

For the secondary outcome in the present study, the use of services (i.e. consultations and hospitalizations) between end of treatment and 1 year follow-up showed no significant difference between the GPM and TAU conditions, with a χ² (1) = 0.58, *p =* 0.446 for consultations and χ² (1) = 2.72, *p =* 0.099 for hospitalizations.

## Discussion

The aim of the present study was to determine the evolution of symptoms of patients with borderline personality disorder over the period ranging between intake (T1) and 16 months after intake (T4), for patients having received brief Good Psychiatric Management compared to treatment as usual. It yielded two main results.

Firstly, the ZAN-BPD scores decreased significantly between randomization and 16 months, for patients receiving good psychiatric management between months 0 and 4. This effect was also found in the TAU condition although the decrease was significantly greater in the GPM condition. This greater decrease could be in part explained by the fact the GPM group had a higher mean score at intake, despite randomization, suggesting that they stood to gain more from the treatment from the start. Conversely, the GPM group had lower scores than the TAU group, at follow-up, suggesting that brief GPM may have had a specific effect the patients’ decrease in self-reported symptoms that lasted past the end of treatment.

To better understand what aspect of BPD related symptoms have decreased, it is important to breakdown the total score into its subsections. This shows us that the decrease in total score comes mainly from two of the four areas that the ZAN-BPD measures., Namely, GPM seems to have a potential added benefit in addressing cognitive and affective aspects of BPD symptomatology compared to TAU. Although the differences between the two groups were not significant for the cognitive subscale, only patients in the GPM conditions saw a significant decrease in scores. This suggests that self-reported identity and dissociative related symptoms decreased, possibly related to the GPM’s focus on expectation and goal setting for which results may take additional time to be felt by patients [10]. For the affective subscale, a lack of significant difference in change between groups may be caused by patients in the GPM condition showing significantly more affective problems at intake. However, this might suggest that the patients in GPM group successfully lowered their perceived affective symptoms such as inappropriate anger, mood reactivity and feelings of emptiness, because of the treatment’s focus on interpersonal hypersensitivity. In turn, learning to mobilize available resources may take an extended period of time for the benefits to be perceived by patients. These results suggest gains continue *after* treatment has ended for the brief GPM group. However, more research is needed to properly identify what is retained from this specific intervention which helps patients continue improving, as studies differ on long-term remittance of symptoms [7]. No significant change was reported on impulsivity related symptoms and relational volatility. Perhaps these aspects require even more time for changes to be experienced and self-reported or perhaps these brief interventions did not target them sufficiently or accurately enough in this study.

Regarding the use of health services, we found that neither group was more likely to use health services in the year following end of treatment. However, our study does not specify whether use of services was directly due to borderline symptoms. This is coherent with Bender et al. [1] which showed that patients who suffer from BPD tend to present with other mental health comorbidities that would lead them to require health care. Perhaps the patients who received brief GPM are now more confident in seeking help at an earlier stage of distress or perhaps patients who received TAU, given their higher drop-out rate, have become less trusting of the health care system. Future researchers may want to further specify causes and types of services used after discharge, as well the number of services used.

A potential reason for the relatively slow improvement could be explained by the overall psychosocial disfunction of patients with BPD [15]. This disorder predicts poor life quality, satisfaction and social functioning. BPD patients may present with a slower improvement in global psychosocial functioning over time than other patients, which may result in new problems that could potentially get in the way of further improvements. One of the added benefits of this treatment is its rapid and inexpensive training [11]. In their study on mentalization based intervention versus structured clinical management for BPD, Bateman and Fonagy [5] showed significant improvement in both conditions, suggesting that low training interventions might be useful in mental health services for BPD. This could potentially lower costs of targeted interventions and make training for these interventions much quicker.

It also important to note that this study stands out for its relatively high inclusion of male patients (33%) and, perhaps, offers a broader understanding of the follow-up effects of brief GPM compared to most studies which are composed of upwards of 80% female patients [16].

Limitations for this follow-up study stemmed mainly from the relatively large amount of missing data. This is due to participants not being able to be reached or not accepting to take part in later time-points of the study. Attrition is relatively severe in most studies conducted on this particular population and we proceeded according to protocol and imputed data, which was needed, and may be introducing biases uncontrolled for. Additionally, this was also the reason why the ZAN-BPD is the only data that we could reliably get at the one-year follow-up. Another limitation was that the GPM group had higher scores than the ones in the TAU group at intake, despite randomization. This makes results more difficult to interpret as the groups had different starting points. Finally, the present study focuses on the symptom changes, and not on functioning. these two interact with one another, in the patient’s achievement of recovery, making functioning important to analyze as well.

## Conclusion

Brief GPM seems to help patients improve more than treatment as usual, one year after the intervention has concluded. It may also potentially have a greater effect in alleviating cognitive and affective related symptoms. This study found that these improvements, however, did not apparently lower the likelihood of using health care services. However, these findings need to be replicated as the high amount of missing data make our claims tentative. Future studies should assess not only symptom change but also functioning, one year after treatment. They could also look into what services are specifically used after treatment and to which extent.

## Data Availability

No datasets were generated or analysed during the current study.
